# Maximizing muscle deoxygenation during interval training in middle-distance runners

**DOI:** 10.1007/s00421-025-05903-1

**Published:** 2025-07-24

**Authors:** Phillip Bellinger, Will Morris, Llion Roberts

**Affiliations:** 1 Griffith Sport Science, Griffith University, Southport, QLD 4215 Australia; 2 School of Allied Health, Sport and Social Work, Griffith University, Southport, QLD 4215 Australia

**Keywords:** Running, NIRS, Muscle oxygenation

## Abstract

The present study aimed to investigate which of two commonly performed running interval sessions elicited the greatest magnitude of and time spent with elevated muscle deoxygenation in trained middle-distance runners. Thirteen trained middle-distance runners (22.4 ± 3.2 y; 63.1 ± 10.9 kg; *n* = 9 males) participated in the study. Subjects completed a field-based incremental running test and two interval sessions. The interval sessions comprised a 6 × 1 km and a 15 × 400 m interval session, both with 1 min passive recovery periods. Both sessions were implemented with the aim of achieving the maximal sustainable pace for each repetition, while mean speed, heart rate, RPE, blood lactate concentration and muscle deoxygenation responses were monitored. Mean speed during the interval repetitions was significantly higher during the 400 m intervals (~ 5.63 ± 0.35 m·s^−1^ vs ~ 5.30 ± 0.28 m·s^−1^; *p* < 0.001). Both the peak magnitude of muscle deoxygenation (absolute difference ± CI 3.42 ± 2.23%; *p* = 0.006) and the time spent with values > 60% peak muscle deoxygenation (83.5 ± 66.4 s; *p* = 0.02) were significantly greater during the 400 m intervals, while the time spent with a heart rate > 90% peak heart rate was significantly longer during the 1 km interval session (570 ± 143, *p* < 0.001). Despite this, there was no difference in RPE, blood lactate concentration or peak heart rate between sessions. These findings suggest that 1 km intervals may preferentially target central physiologic responses while 400 m intervals may elicit greater peripheral physiological responses in trained middle-distance runners.

## Introduction

Elite middle-distance running (i.e., 800 m and 1500 m) competitions range from 1:40.91/1:53.28 (min:s.ms; male/female 800 m world record) and 3:26.00/3:48.68 (male/female 1500 m world record). Maximal exercise within this duration requires a varying blend of energy system contribution and underpinning performance determining physiological and mechanical characteristics (Spencer and Gastin 2001; Duffield et al. 2005a, 2005b; Bellinger et al. 2021a; Bellinger et al. 2021b). Given the diversity in energy metabolism (Spencer and Gastin 2001; Duffield et al. 2005a, 2005b) and performance-determining characteristics (Bellinger et al. 2021a; Bellinger et al. 2021b), a range of central (i.e., maximal cardiac output and hemoglobin mass) and peripheral adaptations (i.e., capillary density and mitochondrial adaptations) may be important when considering the underpinning adaptations that would facilitate training-induced performance improvements in middle-distance running events.

There is now mounting evidence suggesting that high-intensity endurance performance is limited by oxygen transport and the capacity for cellular oxygen utilization (i.e., muscle oxidative capacity) to/within the working muscles (Jacobs et al. 2011; Paquette et al. 2018; Ferri et al. 2012; Zwaard et al. 2018). In this regard, (Ferri et al. 2012) identified that the maximal capacity of muscle to extract O_2_ from the blood (i.e., maximal change in muscle deoxygenation) was strongly associated (*r* = 0.77; *p* < 0.05) with 1500 m running performance in well-trained 1500 m runners (1500 m mean personal best time of 233.3 ± 6.9 s). In support, a recent study by (Paquette et al. 2018) demonstrated that the maximal change in muscle deoxygenation explained a large degree of performance variation in 200-m and 500-/1000-m canoe-kayak time trials in sprint canoe-kayak athletes. These findings suggest that training strategies targeted at improving maximal muscle deoxygenation (higher deoxyhemoglobin responses) may lead to improvements in time trial performance in middle-distance running and canoe-kayak athletes.

Interval training is a critical component of middle-distance training programs (Haugen et al. 2021) given that the highly malleable interval training prescription variables can evoke a wide array of training responses leading to central and peripheral adaptations (Rosenblat et al. 2022), facilitating substantial improvements in time trial performance (Rosenblat et al. 2021). The consensus from the interval training literature concerned with improving VO_2max_ is that the interval training prescription variables should be manipulated to maximize the time spent with VO_2_ above 90% VO_2max_ which may maximize the stimulus for central physiological adaptations to improve VO_2max_ and high-intensity endurance performance (Midgley and Mc Naughton 2006; Buchheit and Laursen 2013a). Akin to maximizing the time spent with VO_2_ above 90% VO_2max_, we could also speculate that maximizing the time spent with a high level of muscle deoxygenation may provide a similar stimulus for peripheral adaptations to improve performance. Two forms of interval training that are commonly used to improve endurance performance are high-intensity interval training (HIT) and sprint interval training (SIT). We define a HIT session as repeated work intervals that are completed in the severe intensity domain and interspersed with recovery periods performed at a low intensity or with complete rest. In contrast, we define a SIT session as repeated maximal efforts of short-duration completed at an extreme-intensity or sprint-type (i.e., all-out) exercise interspersed with recovery periods performed at a low intensity or with complete rest (Bellinger and Minahan 2016). Findings from a recent meta-analysis (Rosenblat et al. 2022) suggested that HIT seems to lead to greater improvements in central factors which may underpin maximal oxygen uptake (i.e., plasma volume, left ventricular mass and maximal stroke volume), while improvements in peripheral factors (i.e., skeletal muscle capillary density and citrate synthase activity) are more likely to result from SIT.

Paquette et al. (Paquette et al. 2019) reported that a SIT session (6 × 30 s all-out sprints w/ 3 min 30 s rest) elicited the longest time at > 90% of maximal muscle deoxygenation compared with 3 HIT sessions (1: 40x[15 s at 115%PPO, 15 s at 30%PPO]; 2: 20x[30 s at 115%PPO, 30 s at 30%PPO]; 3: 6x[1 min at 130%PPO, 3 min rest]) in canoe-kayak athletes. In a follow-up training study implementing HIT (15–30 s efforts at 110% PPO, with 15–30 s active recovery at 50%PPO) or SIT (150–200 m all-out efforts, with 5–6 min passive recovery) (Paquette et al. 2021), the improvements in 1000-m time trial performance were associated with the increases in maximal muscle deoxygenation, suggesting that interval training targeting improving maximal muscle deoxygenation may be important for performance. Nonetheless, there is no research available that has profiled the muscle deoxygenation responses to different interval training sessions in trained middle-distance runners. Therefore, the aim of the present study was to investigate which of two commonly performed running interval sessions elicited the greatest time spent and level magnitude of maximal muscle deoxygenation in trained middle-distance runners.

## Methods

### Participants

A total of 13 well trained middle-distance runners (22.4 ± 3.2 y; 63.1 ± 10.9 kg; *n* = 9 males) participated in the study. All participants in the current study had a minimum of 2 years regular training history for both the 800 m and 1500 m events. The male runners had season-best performance times during outdoor 1500 m track competition of 4:00:62 ± 0:08:90 (3:49:10–4:14:08 s), while the female runners achieved times of 4:53:25 ± 0:10:31 (4:44:15–5:08:10). All runners provided written informed consent prior to participating in this study which was approved by the Griffith University Research Ethics Committee (GU ref no: 2021/519).

### Study design

The current study was a within-subject repeated measures design with the aim of comparing individual muscle deoxygenation responses between two track running specific interval training sessions. The data collection was conducted within the mid-to-late specific preparation phase of the season. Each of the subjects completed a field-based incremental running test and two interval sessions within a 3-week timeframe. Subjects performed a field-based incremental running test to determine their lactate threshold (LT) and lactate turn point (LTP), and estimate their peak incremental running test speed. The experimental sessions comprised a 6 × 1 km interval session and a 15 × 400 m interval session, both with 1 min passive recovery periods between repetitions. Athletes completed both sessions while aiming to achieve the maximal sustainable pace for each repetition, while the acute physiologic responses associated with each interval training session were characterized.

### Field-based incremental running test

Subjects performed a field-based incremental running test which consisted of seven 800 m repetitions on a 400 m synthetic athletics track with repetitions interspersed with 1 min recovery periods during which an earlobe blood sample was taken and analyzed for blood lactate concentration (Lactate Pro 2 device; Arkray inc. Japan). The first six incremental 800 m repetitions were run at a prescribed pace relative to each subject’s recent 10 km personal best times. These times were prescribed through prior experience of the senior researcher (PB) in collaboration with the running coach, whereby the first 6 repetitions were prescribed at paces relative to 10 km personal best times (i.e., stage 1–6: 55%, 65%, 75%, 85%, 95% and 105%), while the seventh stage was a maximal 800 m effort. This protocol was designed with the aim that the sixth 800 m repetition resulted in a blood lactate concentration of ~ 4–6 mmol·L^−1^, while the final stage provided an opportunity to achieve a peak blood lactate concentration and an estimate of peak incremental running test speed. The LT was determined as the running speed of the stage preceding a blood lactate concentration of > 0.4 mmol·L^−1^ above resting values, while the LTP was quantified from the modified D-max method (Bishop et al. 1998).

### Interval training sessions

The subjects completed two different interval sessions on the athletics track in a randomized order. There were 7 days between all sessions, with the same type and content of training performed in the 3 days before each session. One interval session consisted of 6 × 1 km repetitions, while the other interval session consisted of 15 × 400 m repetitions. There were 1 min passive recovery periods between all repetitions. Subjects were instructed to attempt to maintain the highest average running velocity they could across each effort of each interval session. Prior to completing the session, all subjects completed a standardized warmup that consisted of a 10-min self-paced jog and six repetitions of 10-s strides with a walk-back recovery (∼60 s). Subjects also completed a longer 150 m stride. The warm-up procedures were followed by 15 min of recovery. During the recovery period the ischemia/hyperaemia calibration was performed (see details below) and subjects were permitted to perform another 2–3 short strides prior to the first repetition. The subjects were required to provide a rating of perceived exertion (RPE; 6–20) and an earlobe blood sample to determine blood lactate concentration following the completion of every second repetition in the 1 km session (i.e., set 1 comprises repetition 1 and 2; 3 sets in total) and every fifth repetition in the 400 m session (i.e., repetition 1–5 comprises the first set; 3 sets in total), while heart rate was recorded continuously via an M430 running watch (Polar, Kempele, Finland) and chest strap (H10, Polar Electro, Oy, Finland). Peak heart rate was determined as the highest heart rate achieved during a repetition or recovery period, while the time spent with a heart rate value > 90% peak heart rate was also recorded. Muscle deoxygenation responses were monitored using a portable, wireless near-infrared spectroscopy (NIRS) monitor (PortaMon, Artinis Medical Systems BV).

### Muscle deoxygenation

During each interval training session, a portable NIRS monitor (PortaMon, Artinis Medical Systems BV) was placed on the medial head of the gastrocnemius muscle (Bellinger et al. 2019) prior to completing the warmup. This muscle was chosen due to the ease of performing the ischemic calibration as well as the importance of the gastrocnemius to locomotion in running. Subjects adopted a supine position with both legs extended and the NIRS device was placed on the midline of the belly of the medial head of the gastrocnemius muscle and secured with bi-adhesive tape and covered with non-stick opaque crepe bandages. The PortaMon is a compact (83 × 52 × 20 mm), lightweight (84 g) NIRS system with a dual wavelength (760 and 850 nm), continuous wave system, containing three pairs of Light-emitting diodes with a source–detector spacing of 30, 35, and 40 mm. The device simultaneously uses the modified Beer–Lambert law and spatially resolved spectroscopy methods to calculate the absolute concentration of tissue oxyhemoglobin, deoxyhemoglobin, and total hemoglobin (McManus et al. 2018). While all parameters were measured, we chose to focus on muscle deoxyhemoglobin responses as this is thought to be insensitive to blood volume changes during exercise (Blasi et al. 1993). An ischemia/hyperaemia calibration was performed to normalize the NIRS signals using a wireless blood flow restriction device (Airbands, VALD Performance, Brisbane, Australia) placed proximal to the NIRS optodes and above the knee joint line. The cuff was inflated for ~ 10 min (range; 7–13 min at ~ 260 mmHg), until a stable plateau in deoxyhemoglobin existed, and then immediately deflated allowing a 1–3 min period of hyperaemia. This calibration was used to scale the NIRS signals to this range, whereby the deoxygenation signal had a maximum which was allocated 100% corresponding to the plateau during the ischemic period and a minimum which was allocated 0% corresponding to the lowest value in the hyperaemia period. NIRS data were acquired at 10 Hz and exported as a.csv file. To reduce the noise created by movement, we applied a moving average with a window of 5 s and treated the deoxygenation signal with a smooth spline filter (Rodriguez et al. 2018) using GraphPad Prism software (version 10.3.1). Subsequently, we determined the magnitude of maximal muscle deoxygenation relative to the minimum and maximum values obtained from the calibration period, as well as determined the time spent with deoxygenation values > 60% maximal muscle deoxygenation. We chose > 60% maximal muscle deoxygenation as a muscle deoxygenation threshold, as all subjects elicited a maximal muscle deoxygenation value of at least 60% in all training sessions. Skinfold thickness was measured at the belly of the medial gastrocnemius muscle using a skinfold caliper (Harpenden Ltd.), whereby skinfold thickness (men: 3.3 ± 1.0 mm; women: 7.4 ± 1.4 mm) was substantially less than half the distance between the emitter and the detector in every subject.

### Statistical analysis

Statistical analyses were carried out using “The Jamovi project (2021)” (Jamovi Version 1.6 for Windows) with data are presented as means ± standard deviations, as well as absolute difference between sessions ± 95% confidence intervals (CI). Mean speed, RPE and blood lactate concentration were compared using a two-way ANOVA, with Tukey post hoc comparisons used to determine differences between repetitions. Paired T tests were used to compare absolute and relative peak heart rate, the magnitude of maximal muscle deoxygenation and the time spent with deoxygenation and heart rate values above thresholds between sessions. Significance was set at P < 0.05. The effect size (*d*) statistic with a 95% confidence interval (CI) was also calculated to assess the magnitude of difference between interval training sessions. The magnitude of difference was classified as small 0.2–0.6, moderate 0.6–1.2, large 1.2–2.0, and very large 2.0–4.0 (Batterham and Hopkins 2006).

## Results

### Field-based incremental running test

Table 1 displays the mean and range of the LT, LTP and peak incremental running speed results from the field-based incremental running test.

**Table 1:** Results from the field-based incremental running test

Variable	Mean ± SD
LT speed (m·s^−1^)	4.14 ± 0.32
LT absolute heart rate (beats·min^−1^)	164 ± 11
LT relative heart rate (% peak heart rate)	84.8 ± 4.50
LTP speed (m·s^−1^)	4.99 ± 0.30
LTP absolute heart rate (beats·min^−1^)	178 ± 12
LTP relative heart rate (% peak heart rate)	92.2 ± 4.08
Peak incremental running speed (m·s^−1^)	6.08 ± 0.40
Peak heart rate (beats·min^−1^)	194 ± 12

### Comparison between interval sessions

**Fig. 1:**
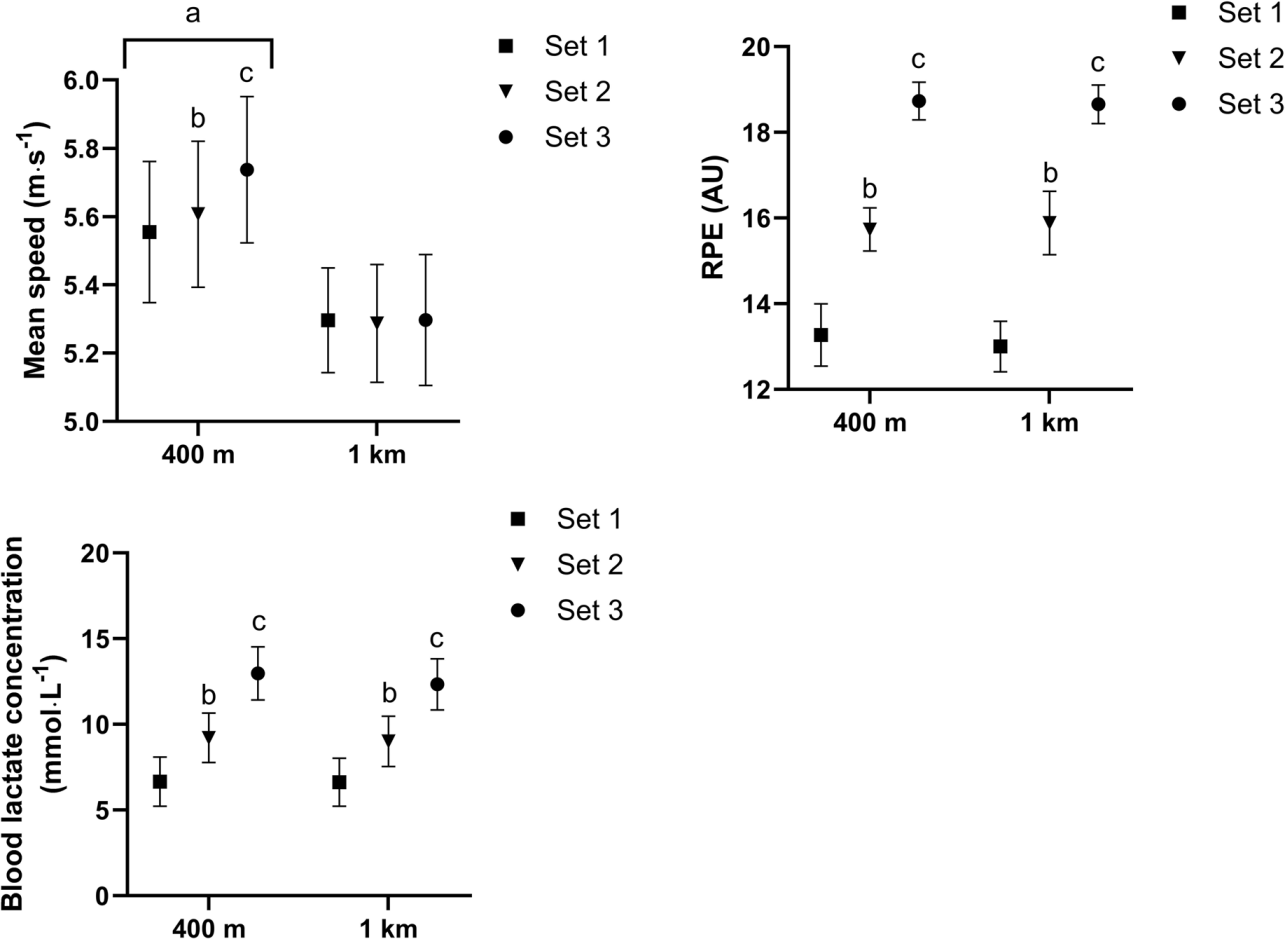
Mean speed (a), RPE (b) and blood lactate concentration (c) for each set (i.e., every second or fifth repetition for the 1 km and 400 m session, respectively). a = significantly different from corresponding set in 1 km session. b = significantly different from first set. c = significantly different from first and second set

Mean speed was significantly higher during the 400 m intervals (session comparison; *p* < 0.001; Fig. 1) when comparing the mean speed from repetitions in set 1 (400 m intervals 5.55 ± 0.34 m·s^−1^; 1 km intervals 5.30 ± 0.25 m·s^−1^; absolute difference ± CI, 0.26 ± 0.09 m·s^−1^; ES = 0.87; *p* < 0.001), set 2 (400 m intervals 5.61 ± 0.35 m·s^−1^; 1 km intervals 5.29 ± 0.29 m·s^−1^; absolute difference ± CI, 0.32 ± 0.08 m·s^−1^; ES = 1.0; *p* < 0.001) and set 3 (400 m intervals 5.74 ± 0.35 m·s^−1^; 1 km intervals 5.30 ± 0.32 m·s^−1^; absolute difference ± CI, 0.44 ± 0.10 m·s^−1^; ES = 1.31; *p* < 0.001). Mean speed also increased across the session during the 400 m intervals, while this did not occur in the 1 km session (session x time; *p* < 0.001; Fig. 1).

There was no difference in RPE between sessions at any time point (session comparison; *p* = 0.822) and RPE increased to a similar extent across both sessions (session x time; *p* = 0.430), whereby RPE increased after set 1 (400 m intervals 13.3 ± 1.20; 1 km intervals 13.0 ± 1.00; *p* < 0.001), set 2 (400 m intervals 15.7 ± 0.83; 1 km intervals 15.9 ± 1.23; *p* < 0.001) and set 3 (400 m intervals 18.7 ± 0.73; 1 km intervals 18.7 ± 0.75; *p* < 0.001) in both sessions.

There was no difference in blood lactate concentration between sessions at any time point (session comparison; p = 0.338) and blood lactate concentration increased to a similar extent across both sessions (session x time; p = 0.234), whereby blood lactate concentration increased after set 1 (400 m intervals 6.66 ± 2.38 mmol·L^−1^; 1 km intervals 6.62 ± 2.32 mmol·L^−1^), set 2 (400 m intervals 9.22 ± 2.39 mmol·L^−1^; 1 km intervals 9.01 ± 2.44 mmol·L^−1^) and set 3 (400 m intervals 12.97 ± 2.56 mmol·L^−1^; 1 km intervals 12.34 ± 2.47 mmol·L^−1^) in both sessions.

**Fig. 2:**
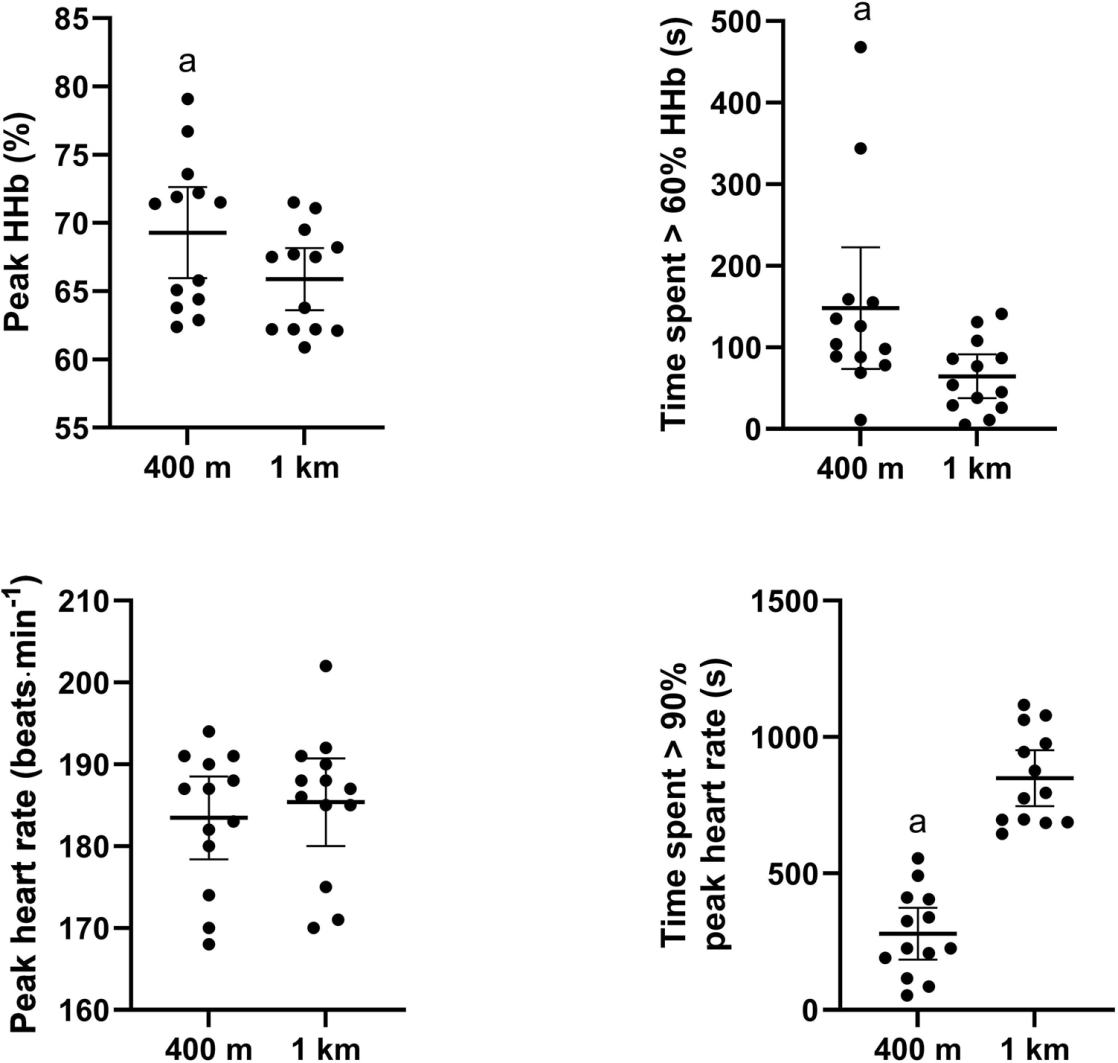
The peak magnitude of muscle deoxygenation (A), time spent with values > 60% muscle deoxygenation (B), peak heart rate (C) and time spent with a heart rate > 90% peak heart rate (D) throughout each session. a = Significantly different from 1 km session

**Fig. 3:**
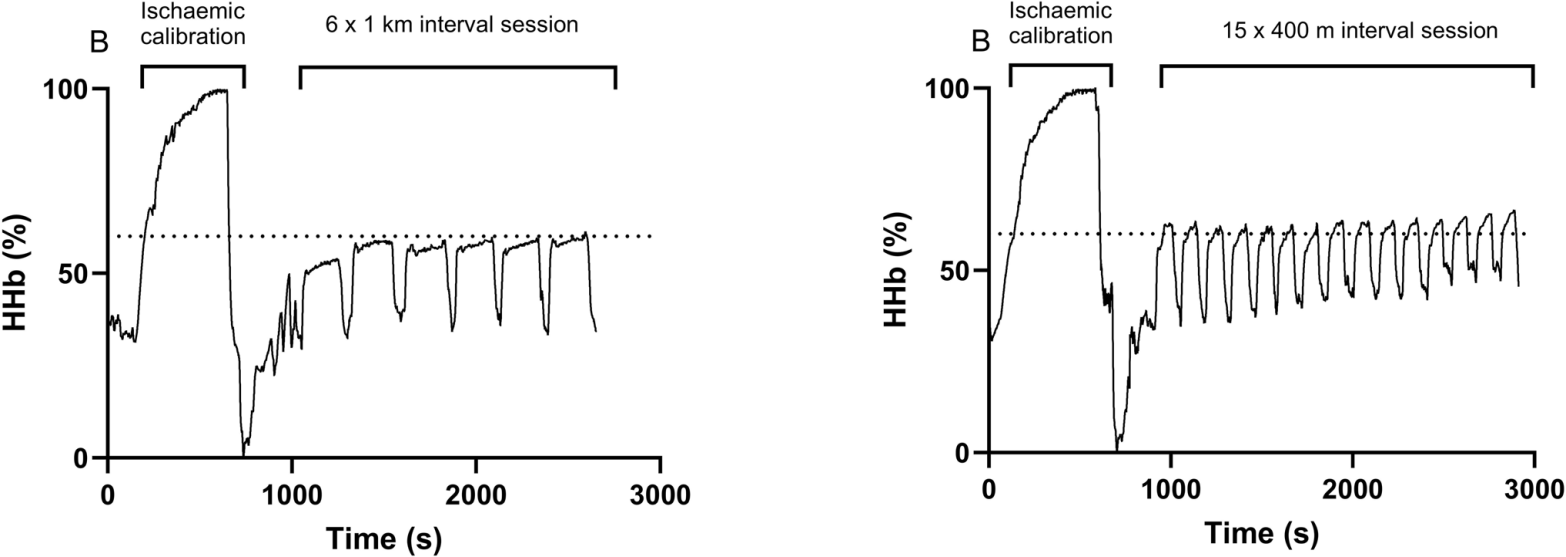
An example of the muscle deoxygenation responses for the 1 km (a) and 400 m (b) session. An ischemia/hyperaemia calibration was performed prior to each session to normalize the NIRS signals, whereby the deoxygenation signal had a maximum which was allocated 100% corresponding to the plateau during the ischemic period and a minimum which was allocated 0% corresponding to the lowest value in the hyperaemia period

Both the peak magnitude of muscle deoxygenation (400 m intervals 69.3 ± 5.53%; 1 km intervals 65.9 ± 3.76%; absolute difference ± CI, 3.42 ± 2.23%; ES = 0.74; p = 0.006) and the time spent with values > 60% muscle deoxygenation (400 m intervals 148.0 ± 123.5 s; 1 km intervals 64.5 ± 44.4 s; 83.5 ± 66.4 s; ES = 1.0; *p* = 0.02) were significantly greater during the 400 m intervals compared to the 1 km intervals (Fig. 2). In contrast, time spent with a heart rate > 90% peak heart rate was significantly longer during the 1 km interval session (849 ± 169 s) compared to the 400 m interval session (279 ± 157 s, 570 ± 143, ES = 3.49; *p* < 0.001). There was no significant difference in peak heart rate (400 m intervals 183 ± 8 beats·min^−1^; 1 km intervals 185 ± 9 beats·min^−1^; 2 ± 4 beats·min^−1^; ES = 0.22; *p* = 0.092) or relative peak heart rate (400 m intervals 94.9 ± 2.94%; 1 km intervals 95.9 ± 3.05%; 1.0 ± 1.77%; ES = 0.3; *p* = 0.104) between sessions (Fig. 3).

## Discussion

The present study is the first to assess the NIRS-derived measurements of muscle deoxygenation during two different field-based track running interval running sessions in well-trained middle-distance runners. The key findings were that 400 m intervals were performed at a higher mean speed and featured a greater peak muscle deoxygenation and a greater time spent > 60% maximal muscle deoxygenation when compared to 1 km intervals. In contrast, there was a greater time spent at  > 90% peak heart rate during the 1 km intervals. Despite these differences between sessions, there was no significant difference in peak heart rate, blood lactate concentration or RPE scores. These findings may have important implications given that peripheral mechanisms are important for middle-distance running performance, and training strategies that target peripheral adaptations may maximize improvements in performance.

The key finding of the present study was that 400 m running intervals performed at a higher mean speed, elicited a greater peak muscle deoxygenation and a greater time spent > 60% maximal muscle deoxygenation when compared to 1 km running intervals. Given the extensive number of training prescription variables that can be manipulated (Buchheit and Laursen 2013a), it is difficult to directly compare these findings with other studies that have characterized the muscle deoxygenation responses to different interval sessions. Nonetheless, the findings from the present study agree with some (Paquette et al. 2019), but not all literature (Zafeiridis et al. 2015) that has characterized the muscle deoxygenation responses to different interval training approaches. For example, Zafeiridis et al. (Zafeiridis et al. 2015) reported that peak and average muscle deoxygenation were not different between short (30 s of cycling at 110% of the power output corresponding to VO_2max_) and long intervals (2 min cycling at 95% of the power output corresponding to VO_2max_) when both were performed with a 1:1 rest:recovery ratio. In contrast, Paquette et al. (Paquette et al. 2019) reported that peak muscle deoxygenation was greatest in two interval sessions with the highest work rate (i.e., all-out and 130% of peak incremental test power output) and smallest work:recovery ratios, while the all-out interval training session (6 × 30 s all-out sprints; 3 min 30 s rest) produced the longest time at > 90% of maximal muscle deoxygenation. As such, it is clear that the intensity of the work interval as well as the work:recovery ratio are important factors that may dictate the muscle deoxygenation responses to a given interval training prescription. Of course, it should also be noted that the duration of the work interval (Buchheit et al. 2012; Buchheit et al. 2012), as well as the duration (Christmass et al. 1999) and the intensity (Buchheit et al. 2009) of the recovery/rest interval also impact the physiological responses, including the magnitude of muscle deoxygenation.

In the present study, the 1 km interval session resulted in a greater time spent with > 90% peak heart rate compared to shorter intervals. The greater running time equated to ~ 14 min with heart rate elevated > 90% peak heart rate, compared to ~ 5 min in the shorter interval session. While heart rate can certainly not be used as a surrogate measure of VO_2_, time spent with > 90% peak heart rate does seem to reflect similar patterns of the cumulative time spent with VO_2_ elevated > 90% VO_2max_ across different interval sessions (Paquette et al. 2019). As such, we could speculate that the 1 km running intervals in the present study may have also resulted in a greater time spent with VO_2_ elevated > 90% VO_2max_ compared to the 400 m intervals. These findings would be in agreement with previously published research with kayak/canoe (Paquette et al. 2019), cycling (Zafeiridis et al. 2015) and rowing (Faelli et al. 2022) athletes, demonstrating that longer intervals, performed at a lower absolute intensity resulted in a greater time spent with VO_2_ elevated > 90% VO_2max_ compared to shorter intervals, performed at a greater absolute exercise intensity. The key element in these studies (Paquette et al. 2019; Zafeiridis et al. 2015; Faelli et al. 2022), including that of the present is that the interval sessions were performed in a manner that led to comparable perceptions of effort across sessions and/or were performed at the highest sustainable work rate for each interval session prescription. In the present study, the intensity (i.e., mean speed) was higher in the 400 m intervals, whereby the shorter work duration during each repetition and longer total session recovery time reduced the time spent with > 90% peak heart rate. Buchheit and Laursen (Buchheit and Laursen 2013a) suggest a goal of ~ 10 min of cumulative time spent with VO_2_ elevated > 90% VO_2max_ per session in order to elicit central cardiopulmonary adaptations in trained endurance athletes. Therefore, the 1000 m interval approach featured in the present study is in line with these recommendations. Given that we also prescribed a 1 min recovery period in between each repetition, the work:recovery ratio was greater in the 1000 m (~ 3.2:1) compared to the 400 m intervals (1.2:1). In line with Buchheit and Laursen (Buchheit and Laursen 2013a), these data also confirm that intervals with a much greater work:recovery ratio should be preferred when attempting to maximize the time spent with a heart rate and/or VO_2_ > 90% peak heart rate/VO_2peak_.

Subjects were instructed to perform each interval training session with their maximal sustainable intensity across all repetitions, which resulted in a greater mean speed and time spent with a heart rate and muscle deoxygenation levels above certain thresholds for each repetition in the 400 m intervals compared to the 1000 m intervals. Despite observing these differences, there were comparable peak heart rate, percentage of peak heart rate, RPE and blood lactate concentration between sessions. In general, longer interval durations (at a fixed intensity) and higher interval intensities (at a fixed duration) will elicit higher blood lactate concentration, heart rate and perceived exertion. Nonetheless, in the context of the present study, subjects performed each interval training session with their maximal sustainable intensity across all repetitions. Therefore, the reduced duration of the shorter intervals was compensated for by a greater mean speed, which led to similar blood lactate, RPE and peak heart rate responses across the two sessions. It is challenging to compare these findings with other research directly, given that the majority of research implementing interval training prescriptions employ fixed work intensity and/or fixed work duration (Buchheit and Laursen 2013a, 2013b). In contrast, precise external control of intensity in track running prescription is unusual, whereby coaches focus on the manipulation of interval duration (i.e., through distance prescription), rest duration, and number of work intervals (Haugen et al. 2021). Nonetheless, findings from the present study are in agreement with some literature who have reported similar indices of peak heart rate (Zafeiridis et al. 2015), perceived exertion (Seiler and Sjursen 2004) and blood lactate concentration (Seiler and Sjursen 2004) following short and long intervals, when instructed to perform each interval training session with their maximal sustainable intensity across all repetitions.

The present study implemented two training sessions that may be considered typical of middle-distance running prescription. While we matched the perceived execution (i.e., maximal sustainable intensity across all repetitions) and the total distance of each session, the total duration and energy expenditure was not necessarily equivalent. Nonetheless, this is typical of how coaches may prescribe of middle-distance track running sessions. Whether the diverse acute responses to these training sessions would lead to differential chronic training adaptations should these sessions be prescribed frequently across a longer training period, remains to be determined. Given the field-based nature of the present study, we were not able to measure key physiologic characteristics such as VO_2_ or cardiac output. Nonetheless, given the number of portable devices (i.e., Portamon, Moxy, TrainRed etc.) that are available on the market, future research should profile the peripheral responses to a wider range of middle-distance running sessions.

Longer running intervals (i.e., 1 km repetitions) resulted in a greater time spent with > 90% peak heart rate while there were no significant differences in peak heart rate, blood lactate concentration or RPE scores. Shorter running intervals (i.e., 400 m repetitions) were performed at a higher mean speed and featured a greater peak muscle deoxygenation and a greater time spent > 60% maximal muscle deoxygenation when compared to 1 km running intervals. Future research should determine whether these differences in acute responses, result in preferential adaptations following chronic training periods in well-trained middle-distance runners.

## Data Availability

Data generated or analyzed during this study are available from the corresponding author upon reasonable request.
